# Alzheimer's Disease: Exploring the Role of Inflammation and Implications for Treatment

**DOI:** 10.1155/2015/515248

**Published:** 2015-11-17

**Authors:** Mark E. McCaulley, Kira A. Grush

**Affiliations:** Yampa Valley Medical Associates, 940 Central Park Drive, Steamboat Springs, CO 80487, USA

## Abstract

Alzheimer's disease (AD) is a neurodegenerative disorder characterized by both structural abnormalities and inflammation in the brain. While recent research has chiefly focused on the structural changes involved in AD, understanding the pathophysiology and associated inflammation of the AD brain helps to elucidate potential therapeutic and preventative options. By exploring the data supporting an inflammatory etiology of AD, we present a case for the use of existing evidence-based treatments addressing inflammation as promising options for treating and preventing AD. We present data demonstrating tumor necrosis factor alpha association with the inflammation of AD. We also discuss data supporting TNF alpha associated inflammation in traumatic brain injury, stroke, and spinal disc associated radiculopathy. We augment this previously unarticulated concept of a unifying pathophysiology of central nervous system disease, with reports of benefits of TNF alpha inhibition in many hundreds of patients with those diseases, including AD. We also assess the pathophysiologic and clinical trial evidence supporting the role of other inflammation resolving treatments in AD. In aggregate, the data from the several potentially effective therapeutic and preventative options contained within this report presents a clearer picture of next steps needed in research of treatment alternatives.

## 1. Introduction

Alzheimer's disease (AD) is both a structural and an inflammatory condition. The following paper describes new ways of viewing brain pathophysiology including features AD shares with other brain diseases. We present several evidence-based means of utilizing existing medications to effect both etiologic and symptomatic improvement of AD. The information contained in this paper will hopefully lead to additional research and, where indicated, clinical trials to further the promising research results seen to date and confirm or refute the effectiveness of the methods described.

## 2. Inflammation in the Nervous System in AD

The structural changes involved in AD have received considerable attention for over two decades. Beta amyloid and tau proteins in tangles and clusters are well recognized as common findings in AD brains. The discovery of accumulation of amyloid-beta peptides (A*β*) in “senile plaques” (SPs) of AD brains gave rise to the amyloid hypothesis, which describes an accumulation of A*β* in the brain that triggers a neurochemical cascade harming both neuronic and synaptic function leading to the cognitive deficits seen in AD.

While the amyloid hypothesis has been the primary paradigm for over two decades of AD research, if it were to accurately explain the pathophysiology of AD, one would expect successful clinical treatment trials targeting the elimination of A*β* accumulation on the central nervous system of AD patients to be effective in alleviating symptoms and/or reversing the condition. Morris et al. summarize well that the results of clinical treatment addressing structural malformations in AD have not had that effect: in fact, so far, anti-A*β* clinical efforts have largely failed to meet primary clinical endpoints and, in some cases, have actually worsened dementia [1].

Although the research investigating the efficacy of the amyloid hypothesis in describing the pathophysiology of AD over the past two decades has shown a correlation between AD and A*β* plaques, causal suggestions are inconsistent and have eroded over time. The premise of the amyloid hypothesis is based on the assertion that A*β* accumulation begins the cascade leading to the symptoms of AD. In 2014, Drachman summarized evidence supporting A*β* accumulation as a downstream effect of an alternate causal process [2]. Some of the most promising research in AD pathophysiology supports an altogether different causal factor: inflammation.

In 1975, Ishii et al. reported electron micrographs showing localization of immunoglobulins in the amyloid fibrils of SPs [3]. In 1984, the same investigators demonstrated complement components in SPs [4]. Cytokines, complement defense proteins, acute phase reactants, signs of microglial activation with scavenger attack, and other indices of inflammation continue to be shown in the brains of AD patients. Elevated levels of tumor necrosis factor are noted in AD brains as well as in cerebrospinal fluid from AD patients [5].

Genetic assessments of AD risk factors also demonstrate inflammation as a potential first step in AD pathophysiology.* Apolipoprotein E4* (APOE4) is closely tied to innate immunity and remains the strongest known risk factor for sporadic, late-onset AD [6]. APOE mimetics have been successful in treating experimental models of a number of neurological diseases, including AD. Furthermore,* triggering receptor expressed on myeloid cells 2* gene (TREM2), like APOE4, has been shown as a risk factor for AD and is closely tied to innate immunity [7].

While it is clear that inflammation is present in AD brains, it has been more difficult to show whether the inflammation is contributing to the structural aberrations and damage or whether the disordered proteins characteristic of AD may be causing a secondary immune response. In a 1996 article, Rogers et al. summarized the data demonstrating immune system components present in AD brains, as well as the toxicity of those components. Structural abnormalities without immune reactivity do not cause AD. Immune activity may cause AD without other structural problems [8].

## 3. The Blood Brain Barrier in AD

The human brain exists and functions with a degree of immunologic isolation. The blood brain barrier (BBB) chiefly serves this purpose by limiting access of blood-derived products to the CNS. In healthy individuals, the BBB limits the entry into the CNS of A*β* from the serum. The structural changes characterizing AD comprise an accumulation of A*β* that should not otherwise be present in the CNS, indicating a loss of BBB functionality.

While the pathophysiology leading to excessive deposition of A*β* in the AD brain remains unknown, impaired transport and elimination of beta amyloid from the CNS via the BBB may be responsible, at least in part, for this accumulation. In healthy individuals, the efflux of A*β* out of the brain and into the cerebrospinal fluid is regulated by LRP (an LDL receptor regulating transport and metabolism of apoE associated cholesterol) and P-glycoprotein, while the influx of A*β* is controlled by RAGE (receptor for advanced glycation end products). Abnormalities in these pathways are prominent in the BBB in AD [9]. Additional proteins known to play a role in microvascular BBB function include endothelial nitric oxide synthase (eNOS) and vascular endothelial growth factor (VEGF). A 2014 study analyzing the autopsy archives of the superior temporal cortex samples of 15 AD cases and 15 control (nondemented, nonneurologically impaired) cases found a significant negative correlation between SP burden and P-glycoprotein (P-gp), VEGF, and eNOS positive capillaries along with a significant positive correlation between SP burden and the presence of LRP and RAGE proteins. These findings strongly indicate altered BBB function in the pathogenesis of SPs in Alzheimer's brains [10].

In addition to increasing SP load, excessive A*β* in the brain is toxic to the tight junctions (TJ), which are the chief structural components of the BBB. Enhanced permeability of the BBB and disrupted microvessels near SP areas suggests a molecular pathway underlying the interference of BBB integrity [11]. Thus, an inciting event in AD may be an insult to the brain, which, by damaging the BBB, diminishes or eliminates the immunologic privilege of the brain, allowing entry to and exit from the brain of a variety of substances which may allow or promote an inflammatory response. Immunologic vulnerability may be a result of genetic or acquired characteristics (see the human biome below). Known risk factors for AD include (though not limited to) age, history of trauma, vascular abnormalities such as atherosclerosis, and genetic factors such as apoE subtypes [12]. All of these factors represent mechanisms which may impact BBB integrity [13].

The degree to which the damaged BBB with immune hyperresponse in the brain explains AD causation in some, or perhaps most, cases is not clear. However, for the purposes of this discussion, the previous paragraphs do set the stage for the central issues addressed in this paper. Namely, is the inflammation which is characteristic of AD a cause of the neural dysfunction and disability of AD or is that inflammation a byproduct of the structural changes and not a particularly major part of the manifestations of the disease itself? A useful way to answer this question is to examine whether therapies combatting the inflammation of AD offer any clinically useful benefit.

## 4. Nonsteroidal Anti-Inflammatory Drugs

Nonsteroidal anti-inflammatory drugs are commonly used in modern societies. Multiple mechanisms seem to be responsible for their pain and inflammation relieving properties. Given the common usage of NSAIDs, despite a number of potentially toxic reactions, they are logical medications for study in evaluating a potential therapeutic role for limiting inflammation in AD. Activation of the peroxisome proliferator g (PPARg) nuclear transcription factor and inhibition of cyclooxygenase-1 and cyclooxygenase-2 are mechanisms induced by NSAIDs that may decrease brain inflammation, thereby affording a degree of prophylaxis of AD [14–16].

Studies of nonsteroidal anti-inflammatory drug impact in prophylaxis of AD have been mostly suggestive of a favorable impact. A study of NSAID use in a Netherlands population (Rotterdam study) showed a significantly decreased likelihood of AD with increasing use of NSAIDs. This prospective study showed increasing protection with longer periods of use, a pattern which increases the probability of a genuine NSAID effect. Short term use of NSAIDs (one month or less) showed a relative risk of 0.95, whereas long term use (24 months or more of cumulative use) showed a relative risk of 0.20, with a confidence interval of 0.05 to 0.83. A strength of this study is the use of computerized pharmacy records for documentation of NSAID use in a country in which pharmacy records provide near complete information on the utilization of pharmaceutical drugs [17].

In attempting to build on this data, randomized placebo controlled trials were performed, with encouraging results. Alzheimer's disease anti-inflammatory prevention trial, reported in 2011 by Breitner et al., utilized three comparator groups: one was placebo, one utilized celecoxib, and one utilized naproxen. Over the initial study period, both NSAID groups had a disappointing increase in AD incidence. However, over a longer period, manifesting after 2-3 years, the naproxen group experienced a decreased incidence of AD. These results were enhanced by measuring a marker of neurodegeneration, CSF ratios of tau to alpha-beta 1-42. The CSF ratios were consistent with reduced neurodegeneration in the naproxen group, but only after 2 or more years of use [18].

The Breitner et al. trial did confirm and amplify a finding from the previous Rotterdam study. NSAIDs, to be effective in preventing AD, must be given for two or more years. New, from the Breitner trial, is the importance of the type of NSAID. The correct NSAID must be used. The benefits of prophylaxis appear to be restricted to patients prior to developing more severe AD.

A meta-analysis from 2003 in the British Medical Journal (BMJ) of multiple studies of NSAID impacts on AD risk also concluded that longer term use, mostly over 24 months, conferred considerable benefit [19]. Longer term NSAID use, mostly more than 24 months, was associated with a 73% reduction in AD incidence. This risk reduction is in the same range as that reported in the Rotterdam and the Breitner trial.

The NSAID, indomethacin, has unique characteristics impacting the brain. Certain headache types, such as “ice pick headaches,” are so responsive to indomethacin that this type of headache has been renamed “indomethacin responsive headaches.” [20] The exact characteristics responsible for this unique aspect of indomethacin pharmacology are not clear [21]. One might expect, therefore, that indomethacin could be a likely NSAID to favorably impact AD incidence. Based on a shorter (six-month) placebo controlled trial that did show a good degree of benefit from indomethacin in prophylaxing AD [22], a longer term trial, 12 months, double-blind, with a placebo control group, was undertaken utilizing indomethacin. The authors reporting this trial noted that “considerable recruitment problems of participants were encountered, leading to an underpowered study.” Despite a 16% reduction in deterioration in the active group compared to the placebo group and even larger differences in other measurements, the unfortunately worded title suggests lack of benefit, when the converse was true. There were simply too few subjects to demonstrate statistical significance. In fact, this study shows considerable promise in the use of indomethacin for preventing Alzheimer's disease. To again quote the authors, “*this study was underpowered.*” Based on the older Rotterdam study, the Breitner report, and the BMJ meta-analysis, a longer period of observation and treatment would have been more likely to show a robust effect. Other useful data from this trial include the relatively minor side effects of indomethacin such as transiently elevated creatinine levels and blood pressure. A 16% reduction in AD incidence in an appropriately sized study is not trivial in the context of a disease of such severity and prevalence. Should this trial have extended beyond one year, to two or more years, one would expect a greater likelihood of efficacy in the indomethacin group.* Absence of proof does not equal proof of absence* [23].

Aspirin and its potential for prophylaxis of AD have been studied. A large, well done, Swedish study of same sex twins (average age 83.9, age range 80–99) observational in design showed prophylaxis of AD with higher dose aspirin, defined as more than 75 mg aspirin per day [24]. Another Swedish study looked at women at high risk of cardiovascular disease, evaluating the relative risks of dementia associated with aspirin use. It found that aspirin lowered the risk of cognitive decline significantly, at doses of 75 to 150 mg aspirin daily [25]. On the other hand, various studies have shown no aspirin related prophylaxis of dementia and some have shown a worrisome increase in risk of aspirin associated cerebrovascular hemorrhage in AD patients, though not normal patients who were at risk of AD [26–28].

To summarize the various aspirin studies, a reasonable conclusion is that any potential role for aspirin in preventing AD is not clear with various studies giving disparate results. The finding in multiple studies of a considerable incidence of intracranial hemorrhage in AD subjects taking aspirin suggests that caution is warranted in any proposed clinical investigation of aspirin in AD. The infrequent occurrence of intracranial hemorrhage related to aspirin in the physicians' health study of nondemented men (2 per thousand over a five-year interval, compared to 1 per thousand in the placebo treated group) is reassuring in the prevention of AD in a group not yet symptomatic with dementia [29]. The pathophysiology of aspirin associated intracranial hemorrhage in a symptomatic AD cohort is not well defined and could be a useful subject of additional study.

## 5. Tumor Necrosis Factor Alpha in Alzheimer's Disease

Tumor necrosis factor (TNF) is a group of proteins that functions to provide protection from some tumors and infections. TNF also contributes to reparative processes in the central nervous system. TNF alpha is the subset of TNF proteins which appears to be the major cause of TNF induced inflammation. A role for TNF alpha in the pathophysiology of AD is suggested by findings of overexpression of TNF alpha in AD brains, localization studies of TNF alpha in AD, elevated levels of TNF alpha in the CSF and blood of AD patients (as noted above), and multiple demonstrated relationships between TNF alpha and beta amyloid protein and tau protein in AD [30].

Activation of the TNF receptor 1 is required for neuronal cell death as a toxic consequence of beta amyloid protein [31]. TNF alpha inhibits learning by inhibiting long term potentiation, a process critical for memory [32]. TNF alpha is present only at very low, barely detectable levels, in the brain in the basal, noninflamed state, but increases in response to neurologic insults. Brain nerve growth factor (NGF) is a neurotrophic factor, the presence of which is crucial to the development and maintenance of brain cholinergic neurons. TNF alpha regulates the levels of brain NGF with higher levels of TNF alpha decreasing hippocampus NGF levels [33].

Cerebrospinal fluid levels of TNF alpha also tend to be elevated in the brain in other pathological conditions, being released in large quantities by the microglia in those conditions. TNF alpha exerts both cytotoxic and excitotoxic effects, creating what has been called a neuroinflammatory syndrome. Excitotoxicity refers to excessive and/or prolonged activation of excitatory pathways leading to cell death. This process is associated with a number of diseases including ischemia, AD, Parkinson's disease, multiple sclerosis, and amyotrophic lateral sclerosis [34].

Inhibition of TNF alpha has been shown to decrease amyloid plaques and tau phosphorylation in the mouse brain, processes associated with dementia [35]. Parkinson's disease induction in a mouse model was prophylaxed with a novel TNF inhibiting agent complexed with a compound in order to facilitate transport through the blood brain barrier [36].

Most agents that inhibit TNF alpha activity do so with monoclonal antibodies directed at TNF alpha. Etanercept (see below) is a soluble synthetic TNF alpha* receptor* linked to the Fc portion of human IgG1. Etanercept binds to, thereby inactivating, TNF alpha. Some diseases that generally improve with most TNF alpha inhibitors include inflammatory bowel disease such as ulcerative colitis and Crohn's disease (though etanercept is not effective in inflammatory bowel disease), as well as rheumatoid arthritis and psoriasis. Given the apparent role of TNF alpha in the pathogenesis of AD, one might expect that TNF alpha inhibitors could benefit patients with AD. Indeed case reports and clinical trials of TNF alpha inhibition in AD patients demonstrate benefit.

## 6. TNF Alpha Inhibition in AD

A problem with TNF alpha inhibition, via monoclonal antibody treatment or synthetic antibody linked TNF receptors, is that the molecules utilized are large and generally unable to cross the blood brain barrier. Tobinick addressed this difficulty by administering etanercept via “perispinous injection” into the subcutaneous tissues at the C5-6 level of the cervical spine, thereby entering the venous plexus in that area, bypassing the blood brain barrier [37]. A radioisotope PET imaging study demonstrates rapid penetration into a rat brain of isotope tagged etanercept administered through the perispinal method [38]. Tobinick et al. have reported several cases and an uncontrolled series of AD patients treated with perispinal etanercept. Tobinick reports marked and rapid improvement (within minutes) in cognitive function in the etanercept treated patients [39–41]. In support of Tobinick's observations are his multiple publications, consistent clinical outcomes and findings, and publications showing substantially similar results by other investigators. A case report from China documents notable cognitive improvement in an AD patient given infliximab, another anti-TNF drug, by intrathecal injection [42]. An independent investigator summarized the six-month pilot study noted above [41] and added her own clinical observations of a group of three additional patients, whom she observed being treated in clinical practice. All three experienced rapid and easily observable clinical improvement, the treatment and response to it being observed by that investigator, who was not otherwise associated with the treating physicians. The perspective of an outside observer describing the response to perispinal etanercept noted in the observed patients adds a level of credence hard to convey in the more clinically oriented reports [43].

In a recent study, Butchart et al. proposed a peripheral mechanism by which anti-TNF alpha may be effective in AD. In that study, etanercept was administered by subcutaneous injection, peripherally, weekly for 24 weeks. There were benefits in the measured outcomes, and statistical significance was achieved in some measurements but not in others [44]. A randomized phase 1 clinical trial (ClinicalTrials.gov Identifier: NCT01716637) is underway examining etanercept administered via the perispinal route in Alzheimer's patients. This trial is a crossover, open label design with perispinal etanercept at a dose of 25 mg weekly plus nutritional supplements versus nutritional supplements alone being administered for six weeks, followed by a four-week washout period, and then crossing over to the other group. In this way each patient serves as his own control. Planned enrollment was 12 patients. Study completion date is estimated to be September 2015. Hopefully, this trial will add useful information regarding the efficacy of perispinally administered etanercept in AD. We are not aware of randomized placebo controlled clinical trials of perispinal etanercept in treatment of AD.

## 7. Tumor Necrosis Factor Alpha in Stroke and Traumatic Brain Injury

Stroke and traumatic brain injury appear to be associated with abnormal TNF alpha activity. TNF alpha is present in only low concentrations in normal brain tissue. However, both cerebrospinal fluid and blood TNF levels increase after ischemic stroke in humans [45]. After induction of experimentally induced cerebral ischemia in rodents, TNF alpha mRNA increased 2- to 3-fold within 1 hour. An 8-fold increase was observed at 4 hours [46].

Anti-TNF alpha trials demonstrate improved clinical outcomes in experimental and human stroke. An antibody directed against TNF alpha demonstrated improved outcomes in experimental hemorrhagic stroke in rodents [47]. Intravenous etanercept administered to mice resulted in improved functional outcome in experimentally induced focal cerebral ischemia [48]. In a series of 629 human patients, Tobinick et al. report salutary impact of perispinous etanercept in traumatic brain injury and stroke. Improvement was noted even in patients treated over 10 years after the trauma or stroke [49, 50]. Tuttolomondo et al. summarized the evidence of a TNF alpha role in stroke and brain injury and potential benefits of TNF inhibition [51]. It is worth noting that the perispinous as well as intravenous route of administration both produced therapeutic effects in stroke and traumatic brain injury. The efficacy of the systemic route of administration adds additional credence to the usefulness of the perispinous route.

## 8. TNF Alpha in Spinal Disc Disease 

Spinal disc diseases with nerve root irritation and inflammation (radiculopathy) are common clinical problems. Considerable evidence suggests spinal disc disease with radiculopathy is related to abnormal TNF alpha activity.

Igarashi et al. showed that application of TNF to nerve roots in rats produced neuropathy similar to that seen in spinal disc disease [52]. Similarly, Wagner and Myers showed that TNF injection into the nerve roots in an experimental animal model created behavioral changes suggestive of nerve irritation and injury [53]. Tobinick and Britschgi-Davoodifar reported a series of 20 patients with significant, chronic spinal disc disease. This group of patients was given etanercept injection via the perispinous route. The patients experienced “rapid, substantial, and sustained clinical pain reduction.” Although the series had no placebo controlled group and was not blinded, the results achieved a high level of statistical significance [54]. Freeman et al. reported a randomized, placebo controlled double-blind trial of transforaminal epidural injection of etanercept for lumbar disc disease. There was marked improvement in the etanercept group [55].

## 9. TNF Alpha Inhibition Plausibility

A barrier to accepting the concept of TNF as a treatable cause of neuroinflammation is the rapidity of its effect. Most studies of anti-TNF therapy have shown nearly unbelievably quick responses, usually within minutes. For a treatment that reduces inflammation, one would expect hours to days or weeks for the therapeutic benefit to manifest. In contemplating how to explain a reduction of symptoms in ten minutes, we postulate a possible neurotransmitter property of TNF alpha.

## 10. TNF as a Neurotransmitter

Tumor necrosis factor alpha has been shown to be an important regulator of synapse function as well as a regulator of gliotransmission [56, 57]. Elevated levels of TNF alpha cause a rapid increase in excitatory synaptic strength and weakening in inhibitory synaptic strength [58, 59]. The times involved for synaptic transmission were measured in milliseconds [60]. These results suggest that TNF alpha may itself be an excitatory neurotransmitter or an excitatory neuromodulator and by implication that binding TNF alpha would result in rapid resolution of abnormal excitatory neurotransmission. This mechanism could account for the rapid response seen in clinical observations with resolution of various neurologic symptoms in minutes following administration of agents that bind and inactivate TNF alpha.

## 11. TNF Alpha in Diseases of the Brain, Spinal Cord, and Nerve Roots

As noted in the preceding paragraphs, substantial evidence suggests a major role, perhaps even a critical role, of TNF alpha related inflammation, in neurologic disease and injury, at multiple levels in the nervous system. TNF alpha related inflammation appears to amplify injury or disease processes in the central nervous system and at interface of the peripheral and central nervous system at the level of the spinal nerve roots. Supporting the evidence of TNF alpha involvement in the pathophysiology of such neurologic conditions is the considerable benefit from anti-TNF alpha therapies. The identification of neurotransmitter capabilities of TNF alpha supports the plausibility of a rapid effect, easily in the range of the ten-minute onset reported by several investigators. More research is needed to clarify the role of TNF alpha in the conditions noted. In particular, randomized placebo controlled clinical trials are needed to confirm or refute the remarkable results reported to date in perispinous administration of etanercept in AD. Markers are needed for denoting brain specific inflammation. The blood levels of TNF alpha and potentially other inflammatory mediators need to be assessed for their utility in diagnosing AD and potentially monitoring treatment. Research is also warranted in other neurologic conditions, such as multiple sclerosis, Parkinson's disease, amyotrophic lateral sclerosis, and others, seeking the degree to which TNF alpha related inflammation may also play a causative role in those conditions. In addition, consideration needs to be directed at the potential for other inflammatory mediators to be involved in neuropathology. TNF alpha is one compound out of many compounds expressed as a part of inflammatory pathways. Elucidation of the potential roles of other such compounds could yield greater understanding of the mechanisms of neuroinflammation, potentially directing attention to even more efficacious and specific treatments.

## 12. Corticosteroids

Corticosteroids such as prednisone, hydrocortisone, methylprednisolone, and dexamethasone are widely used in medicine. While corticosteroids suppress inflammation in various parts of the body, they create risks of several important side effects. If anti-inflammation treatment or prophylaxis of AD is a valid and useful concept, one might expect corticosteroid therapy to be beneficial even at the risk of serious side effects. In an autopsy study of 694 brains of subjects found to have pathologic findings typical of AD or vascular dementia, use of corticosteroid therapy was associated with approximately 50% fewer neuritic plaques and neurofibrillary tangles [61]. Alisky presented evidence of benefit of high dose but not low dose corticosteroid treatment of AD in a 2008 paper [62]. Since high dose corticosteroid treatment carries severe toxicities, Alisky suggested consideration of intrathecal corticosteroid administration. In a study of twins, a substantial and statistically significant risk reduction for AD was associated with prior use of corticosteroids [63]. However, a placebo controlled randomized trial of low dose prednisone in AD did not find any evidence of benefit [64]. Additionally, a study of elderly humans demonstrated that elevated cortisol levels were associated with memory defects and atrophy of the hippocampus, a structure critical for memory [65].

## 13. Antihistamines

Antihistamines are substances which dampen allergy and other immunologic reactions. Although there is rationale in basic science for potential benefit of antihistamines in AD prevention and treatment, clinical trials and epidemiologic investigations have yielded mixed results [66, 67]. Antihistamines have anticholinergic properties. The deficiency of acetylcholine in AD brains appears to be one of the central mechanisms by which AD progresses [68]. Hence one might expect some worsening of AD risk through anticholinergic mechanisms inherent in antihistamines. The immune system modulation by antihistamines might improve AD risk. These opposing mechanisms could account for the conflicting research findings of the effects of antihistamines in AD.

### 13.1. Drugs for Multiple Sclerosis with Potential Activity in AD 

#### 13.1.1. Pirfenidone

Pirfenidone is an inhibitor of TNF alpha production and of its pharmacologic and toxicologic activity. Being a small molecule, pirfenidone easily crosses the blood brain barrier. After initial promising results in two open label studies in secondary progressive multiple sclerosis, pirfenidone was then also studied in a phase 2 randomized, placebo controlled trial in secondary progressive multiple sclerosis [69–71]. Twenty-five patients were randomized to pirfenidone and 18 to placebo. Compared to placebo, the pirfenidone group experienced significantly improved Scripps Neurological Rating Scale scores starting at one month and persisting for the 12-month duration of the trial, decreased numbers of relapses, and markedly improved bladder function. Blood tests remained normal in both groups. Toxicities ascribed to pirfenidone were mild and well tolerated. Because pirfenidone has antifibrotic properties, it was subsequently studied and found to be effective in pulmonary fibrosis. Pirfenidone was approved by the US FDA in 2014 for treatment of pulmonary fibrosis, again being well tolerated with mild side effects. No further trials of pirfenidone in multiple sclerosis have been performed. Pirfenidone has not been studied in AD.

Given the anti-TNF activity of pirfenidone and its apparent efficacy and safety in multiple sclerosis, as well as its ability to cross the blood brain barrier, it may be candidate drug to study in an AD population. In addition, the benefits of pirfenidone in multiple sclerosis suggest a possible role for TNF alpha in the etiology of multiple sclerosis, as well as potential benefit of other TNF inhibiting agents in its treatment.

## 14. Anti-Alpha-4 Integrin Therapy

Lymphocytes are often involved in diseases of the central nervous system, as well as other areas of the body. To migrate from the circulation through vascular structures, lymphocytes adhere to vascular endothelial cells in the walls of blood vessels. Alpha-4 integrin is expressed on the surface of inflammatory lymphocytes and is thought to play a critical role in the ability of lymphocytes to adhere to the vascular wall. Natalizumab is a monoclonal antibody directed at alpha-4 integrins. Natalizumab is FDA approved for treatment of multiple sclerosis as well as Crohn's disease. As a treatment for multiple sclerosis, natalizumab is perhaps the most effective available treatment [72–74]. Unfortunately, a common virus, JC virus (JCV), may reactivate in a small subset of treated patients, causing a brain disease known as progressive multifocal leukoencephalopathy (PML). Though occurring in less than 1% of patients treated with natalizumab, PML is a devastating disease, usually fatal or a cause of severe and permanent disability [75]. Patients who do not show antibodies directed at JCV are thought to be noninfected with JCV and have little or no risk of PML [76]. Although not studied in AD, natalizumab treatment was associated with improved cognition and decreased fatigue in multiple sclerosis patients [77]. The improvement associated with natalizumab therapy in multiple sclerosis patients and the activity of natalizumab in the brain suggest the potential utility of studying natalizumab for a possible role in AD therapy, likely confining its use to patients who test negative for antibodies to JC virus. 60–80% of a North American population would be expected to test positive for JC virus antibodies. Natalizumab's potential for reactivating JCV should add a cautionary note to considerations of immune modifying therapies in general and, pertaining to this paper, in AD in particular.

## 15. Other Agents

The above discussion presents a far from complete picture of immune modulating possibilities in treating or preventing AD. Pentoxifylline and thalidomide are two agents discussed in some reports of potential AD risk reducing medications [78]. There are abundant other agents in different classes of medications which impact immunity in ways that could impact brain inflammation. These agents may be existing drugs that range from respiratory disease treatments such as leukotriene inhibitors to treatments for connective tissue diseases such as systemic lupus or rheumatoid arthritis, to inhibitors of organ rejection, to cancer chemotherapy, to psychopharmaceuticals. New agents will arise from the increasing capability of biopharmaceutical researchers to produce monoclonal antibodies targeting almost any step of most biochemical/physiologic and inflammatory pathways. This capability has created and will create almost limitless possible compounds that may be the “magic bullets” that halt the inflammatory pathophysiology responsible for AD and multiple other diseases.

## 16. The Human Biome

The human biome, referring to microbial and other organisms that live in and on us, may hold clues in understanding and treating inflammatory conditions. Many authors have expressed concern that components of the human biome, with which humanity has evolved for millennia, are now missing.

The most compelling data suggesting a role of biome depletion in AD pathophysiology comes from the hygiene hypothesis. Inadequate training of the immune system through limited exposures to adequate numbers and types of microorganisms appears to result in decreased numbers and poorer function of T reg lymphocytes [79–81]. In experimental T reg deficiency, diseases of profound autoimmunity rapidly afflict the involved subjects [82].

Molly Fox and colleagues from Cambridge University studied the prevalence of AD in populations throughout the world as related to levels of hygiene. The results were adjusted for age [83]. There was a striking relationship between hygiene levels and risk of AD. The more hygienic populations, such as North Americans and North Europeans, had a markedly higher risk of AD than the populations with lowest hygiene levels. There was about a 10-fold risk difference. Societies with intermediate hygiene levels had intermediate AD risk. In another report, in a study of Latin America, India, and China [84], prevalence of dementia was found to be significantly lower in lower socioeconomic countries and portions of countries compared to higher socioeconomic areas. Incidence of AD was significantly lower in an Indian (not native American) population compared with a comparable population in Pennsylvania [85].

Although mouth dwelling organisms may be considered a part of the biome, infection of the gingival tissues, called periodontitis, has been hypothesized as a potential risk factor for AD. According to this hypothesis, gingival inflammation related to chronic, long term, ongoing gingival infection may stimulate immune mechanisms that result in increased risk for AD [86, 87].

One may be tempted to attribute the hygiene associated increased AD risk to lack of an optimal biome, which is consistent with the data. In fact the studies noted above do provide evidence that the modern biome is a factor in the explosion of AD in modern societies. However there is a multitude of other factors which differentiate high from low hygiene populations, such as diet, exercise, and various chemical and biologic exposures. These factors need to also be considered as possible causes of the AD risk difference in various societies.

## 17. MSF (Methanesulfonyl Fluoride)

AD is associated with the loss of acetylcholine effect in the brain. Though not specifically associated with inflammation, this loss of acetylcholine effect is thought to be one of the chief causes of disability in AD brains. Additionally, the loss of acetylcholine effect also contributes to further structural damage. It is important to address acetylcholine levels in order to potentiate the benefit which could accrue from treatment of inflammation. An example of an acetylcholine augmenting medication is that of MSF, methanesulfonyl fluoride, which works in a similar fashion to the FDA approved cholinesterase inhibitors well known as treatments for AD, such as donepezil. MSF, being a smaller molecule, inhibits brain acetylcholinesterase (AChE) more effectively, with fewer systemic toxicities, than the approved medications. Working through irreversible AChE inhibition, the use of MSF takes advantage of very slow de novo resynthesis of AChE in the brain compared to the periphery. The therapeutic benefits of MSF appear to exceed those of the currently approved medications. However, as an existing chemical, with limited means of patent protection, bringing MSF to market has proven excruciatingly difficult, so far impossible [88, 89].

## 18. Conclusion

Alzheimer's disease is an inflammatory, structural, and functional disease of the brain. This report presents evidence supporting a unified pathophysiology of inflammation in AD, traumatic brain injury, stroke, and spinal disc disease. We enumerate a spectrum of potentially effective therapeutic and preventative options for AD and other diseases of the CNS with already existing medications and agents which target inflammatory processes. To date, clinical trials of drugs that modify the inflammatory response in AD have not been conclusive in modifying cognitive decline. Randomized, controlled clinical trials are needed. Factors inducing the inflammatory response associated with AD remain largely unknown. The personal misery AD creates in those people afflicted with it is profound. The increased frequency of Alzheimer's disease in older age groups and the general aging of the population in technologically advanced societies have multiplied to create a pending catastrophic increase in the number of afflicted patients. Numbers of Alzheimer's afflicted patients are expected to nearly triple in the US by 2050. The resources currently required for care of Alzheimer's patients, already a societal burden, are far lower than the expected resource requirements just a few years hence. We hope this report will lead to research to confirm or refute the apparent usefulness of these medications and agents to limit the disability, suffering, and premature deaths caused by this tragic disease.
